# Imitation in Chinese Preschool Children: Influence of Prior Self-Experience and Pedagogical Cues on the Imitation of Novel Acts in a Non-Western Culture

**DOI:** 10.3389/fpsyg.2020.00662

**Published:** 2020-04-15

**Authors:** Zhidan Wang, Andrew N. Meltzoff

**Affiliations:** ^1^School of Education Science, Jiangsu Normal University, Xuzhou, China; ^2^Institute for Learning & Brain Sciences, University of Washington, Seattle, WA, United States

**Keywords:** Chinese culture, imitation, preschool children, social cognition, observational learning, social learning, novel behaviors, over-imitation

## Abstract

Both prior experience and pedagogical cues modulate Western children’s imitation. However, these factors have not been systematically explored together within a single study. This paper explored how these factors individually and together influence imitation using 4-year-old children born and reared in mainland China (*N* = 210)—a country that contains almost one-fifth of the world’s population, and in which childhood imitation is under-studied using experimental methodology. The behavior of children in this culture is of special interest to theory because traditional East Asian culture places high value on conformity and fitting in with the group. Thus, high-fidelity imitation is emphasized in the local culture. This value, practice, or norm may be recognized by children at a young age and influence their imitative performance. In this study, we crossed prior self-experience and pedagogical cues, yielding four demonstration groups in addition to a control group. This design allowed us to investigate the degree to which Chinese preschoolers’ imitation was modulated by the two experimental factors. High-fidelity imitation was significantly modulated by prior self-experience but not by pedagogical cues, as measured by the number of novel acts imitated and also the serial order of these acts. This study (i) expands our understanding of factors that modulate imitation of novel behaviors in preschoolers and (ii) contributes to efforts to broaden research beyond Western societies to enrich our theories, particularly regarding social learning and imitation. Imitation is a key mechanism in the acquisition of culturally appropriate behaviors, mannerisms, and norms but who, what, and when children imitate is malleable. This study points to both cross-cultural invariants and variations to provide a fuller picture of the scope and functions of childhood imitation.

## Introduction

Imitation plays an important role in early social-cognitive development (e.g., Legare, 2017; Meltzoff and Marshall, 2018; Tomasello, 2019). Preschool children are adept at imitating novel acts that they see others perform, including using objects in particular ways, moving their bodies, the serial order in which behaviors are performed, and employing tools (e.g., Want and Harris, 2001; Carpenter et al., 2002; Nielsen, 2006; Nadel, 2014; Subiaul et al., 2015; Loucks et al., 2017). This human proclivity to imitate the specific details of others’ behavior may have played evolutionary roles in (i) binding early humans to their *social* groups and (ii) supporting the diffusion of new *instrumental* behaviors from one person to another (e.g., how to create and use a stone tool or build a fire). In modern humans, imitative learning continues to play a prominent role in the rapid and flexible transfer of non-linguistic information from caretakers to children. Meltzoff et al. (2009) summarized the value of imitation for child learning: It is faster than Skinnerian shaping and conditioning by caretakers; safer than trial-and-error learning by the child; and more responsive to the social-environmental context than children’s individual invention.

The capacity to imitate *novel* behavior is especially important if imitation is to fulfill its theorized role in development. If children were constrained to duplicating only familiar acts that they had already mastered before watching the model, imitation would not enjoy the status it has in theories of developmental science, pedagogy, and human evolution. If this constraint was in place, children could not learn new social customs, behaviors, rituals, or practices from watching others, nor learn how to use novel tools to achieve instrumental ends. The imitation of novel acts is a Rosetta stone for investigating the nature and functional value of imitation in childhood (Meltzoff, 1988b; Meltzoff and Marshall, 2018).

Imitation is a mechanism for learning new behaviors, but children do not imitate everything they see all the time. Scientists have become increasingly interested in the scope of activities that children duplicate and the factors that *modulate* the expression of imitation. For example, there is a distinction made between re-enacting an outcome achieved by an adult (often dubbed “emulation”) versus imitating the particular means and specific acts used to achieve that end (e.g., Tomasello et al., 1993). In one early investigation, it was shown that 14-month-old children re-enacted not only outcomes, but also the distinctly unusual movements and means used by adults. In this study, Meltzoff (1988a) found that infants would imitate the novel act of turning on a light panel by tapping it with their forehead after seeing an adult perform that novel act. Thus, children imitated the specific act or means demonstrated by the adult even though it was unusual, not causally necessary, and unlikely to occur by chance (the head-touch action did not occur in either of the two control groups tested). Following this report of imitation of a completely novel act, a large range of studies, using different procedures and tasks, has explored children’s proclivity to imitate novelty across different ages and situations.

One prominent line of work has shown that young children will duplicate unusual behaviors when these acts are unnecessary, irrelevant, and even counterproductive for achieving a desirable physical outcome (e.g., Lyons et al., 2007, 2011; McGuigan et al., 2007; Nielsen and Tomaselli, 2010; Hoehl et al., 2014). This tendency has been referred to as “over-imitation” (although this term itself has been questioned, inasmuch as the word “over” might be misleading; the research may be thought of as investigating the imitation of novel acts and the conditions under which children exhibit imitation of such behaviors even when they are not necessary for achieving a physical-instrumental end). In terms of theory, Lyons et al. (2007) originally proposed that “over-imitation” is a manifestation of an automatic and compulsory tendency to imitate in the human child. This process has been dubbed automatic causal encoding (ACE). The ACE claim is based on the observation that children will over-imitate despite being capable of identifying and skipping these irrelevant actions, and even though they will acknowledge that such actions are unnecessary if asked (Lyons et al., 2011). However, a different view about over-imitation is that it is an act of social affiliation between the child and the demonstrator (e.g., Nielsen and Blank, 2011; Over and Carpenter, 2012). A third view is that over-imitation is driven by a motivation to adhere to apparent social norms (Kenward et al., 2011; Kenward, 2012; Keupp et al., 2013). Within this literature it has also been noted that studies examining over-imitation often use arbitrary actions with no obvious cause-effect relation with the outcome (dubbed “causally opaque”), which may influence copying.

Regardless of these theoretical debates about the meaning and motivation of “over-imitation,” other researchers, working from different theoretical orientations have focused on the fact that young children’s imitation of novel acts is not compulsory but rather can be highly selective (e.g., DiYanni and Kelemen, 2008; Williamson et al., 2008; Meltzoff and Williamson, 2013; Yu and Kushnir, 2014). This selectivity has captured the attention of theorists, because it highlights the agentive, active, and interpretive aspects of imitation. In one example, Clegg and Legare (2016) showed that children replicated irrelevant actions demonstrated as part of making a bead necklace (e.g., using each bead to touch forehead) only when the task was coupled with normative framing (e.g., “everyone here always does this”) but not otherwise. This weighs against automaticity and favors the selectivity and modulation of the imitation of novel acts. Similarly, it has been reported that children’s novel- and over-imitation is dampened when the demonstrator is absent (Nielsen and Blank, 2011), is a single peer or a puppet (McGuigan and Robertson, 2015), does not belong to the same assigned group as the child (Schleihauf et al., 2019; Wilks et al., 2019), and is, herself, the target of discrimination or prejudice (Skinner et al., 2017, 2019). This line of work suggests that children are not automatically and blindly copying, but rather that there is agency and selectivity involved. From this perspective, childhood imitation is more properly thought of as malleable, modulated, and related to the interpretive context—as an active choice driven by social-cognitive factors—rather than blind, rote, and uncontrollable; in other words, “children choose whom, when, and what to imitate” (Meltzoff et al., 2009, p. 285; Meltzoff and Marshall, 2018).

Several factors have been postulated to modulate children’s high-fidelity imitation of novel acts. This paper explores two such factors in a systematic way in a sample of preschool children born and raised in China—a culture that contains almost one-fifth of the world’s population and with socialization practices that differ in important ways from Western culture. Comprehensive and generalizable theories of imitation cannot be advanced without knowing more about imitation among children reared in this culture. Claims about childhood imitation in general are incomplete if they do not test or consider imitation in traditional East Asian cultures such as China. One rationale for the current work is to broaden our understanding of factors modulating imitation of novel acts in a non-Western sample. In addition to age, at least two directly manipulable factors have been proposed to modulate children’s high-fidelity imitation of novel acts—one of these focuses on what the child brings to the imitation situation (aspects of their own experience and agency) and the other focuses on what the adult brings (aspects of pedagogy).

### Prior Self-Experience

Results from a series of recent studies have suggested that preschool children’s imitation of novel target acts is influenced by the children’s own *prior self-experience* (Williamson et al., 2008; Williamson and Meltzoff, 2011; Wood et al., 2013; Schleihauf et al., 2018; see also Nielsen et al., 2012). For example, in Williamson et al.’s (2008) study, preschool children were randomly assigned to two prior-experience groups. In one, children had prior self-experience that the goal was easily achievable by them; in the other, children had experience that made the goal difficult to achieve (a trick mechanism made a box easy/hard to open). Following this self-experience, the children saw the adult perform an causally unrelated act (e.g., moving a toggle switch) en route to achieving the goal of opening the box. Results revealed that children who had prior self-experience of easily achieving the goal using their own means were less likely to faithfully imitate the adult’s unusual action. (Children assigned to having difficulty achieving the goal were more likely to imitate the unusual action they saw, seemingly motivated to try something new). The authors theorized that children’s prior self-experience *modulated* children’s proclivity to imitate the unusual acts. The nature of children’s self-experience was postulated to set up “priors” that influenced children’s imitation.

### Pedagogical Cues

A second factor that has been argued to modulate children’s high-fidelity imitation of novel acts is *pedagogical cues* (e.g., adult initiated mutual eye contact, child-directed speech, Csibra and Gergely, 2009) that may indicate that the adult is trying to teach the child (e.g., Nielsen, 2006; Buchsbaum et al., 2011; Király et al., 2013). For example, Király et al. (2013) replicated Meltzoff’s (1988a) head-touch study and reported that 14-month-olds copy the novel, relatively inefficient head-touch act more frequently after observing a communicative model doing this action than after incidentally observing a non-communicative model. The authors proposed that pedagogical cues, such as direct communication and ostensive signals may support children’s imitation. Other researchers have downplayed the necessity of pedagogical cues. In Hoehl et al. (2014) 5-year-olds imitated causally unnecessary actions both when they were modeled by a communicative/pedagogical experimenter and when they were not. In Schmidt et al. (2011), 3-year-olds saw an adult perform a novel action without producing any ostensive cues, and yet the children imitated.

Although a number of experiments have documented the influence of prior experience and/or pedagogical cues in separate studies, to the best of our knowledge no research to date has been designed to explore children’s relative weighing of prior self-experience and pedagogical cues by systematically crossing these factors within the same study in the same age group. Nor have the effects of these two factors been systematically studied in children born and raised outside of traditional Western cultures. Without this work, generalized inferences for developmental theory remain somewhat limited.

### Rationale and Novelty of the Study

We investigated the role of prior self-experience and pedagogical cues on children’s high-fidelity imitation of novel acts. Following the call for scientists to increase the use of participants from outside of Western, Educated, Industrialized, Rich Democratic (dubbed WEIRD) societies (Henrich et al., 2010), we tested preschool children in China—a culture that highlights and values conformity and duplication of the actions of teachers and parents. If the role of prior self-experience generalizes beyond Western cultures, we would predict more high-fidelity imitation of causally irrelevant, novel acts for children who lacked prior routines or habits for manipulating these objects. This is based on the idea that the uncertainty of what to do with the novel object makes children more attuned to adopting the specific acts and techniques demonstrated by the adult.

We also examined the degree to which pedagogical cues affect imitation of novel acts in this same study. This is of interest because it has been established that most Chinese parents tend to interact with young children in a “more authoritarian” manner than do Western parents, expecting more conformity and obedience to cultural ways of doing things (Chao, 1994; Chen et al., 2000; Chang et al., 2003; although within-culture variation certainly also exists, Zhu and Zhang, 2008; Xu et al., 2014). In general, Chinese parents do not readily provide the pedagogical cues described in Western samples (e.g., mutual gaze and parentese) to scaffold and support each step of their children’s learning. In traditional Chinese culture, parents tend to teach their children in a more regimented fashion (Chinese idiom; “*bu gou yan xiao*,” in English, “*not frivolous in talking and joking*”) (Shek, 2002). We hypothesized that although prior self-experience may play a more culturally invariant role in modulating children’s imitation (less certainty about what to do leading to higher reliance on others), pedagogical cues may have little or no influence on high-fidelity imitation in China, because children are not socialized to need, value, or expect this kind of support.

The design of the current study expands the literature in two ways. One potential contribution is that we systematically crossed prior self-experience and pedagogical cues in a study of children in China. To date, only two experimental studies of imitation have been reported from China (Wang et al., 2015; Li et al., 2019). In Wang et al.’s (2015) study, the researchers examined whether children could categorize objects by weight after observing the adult’s demonstration of such sorting behavior, and the results showed that 4-year-olds, but not 3-year-olds, imitated the categorization rule (sort visually identical objects by the hidden property of weight, which might have been interpreted by children as a social norm or convention). In Li et al.’s (2019) study, the researchers reported that children imitated an ingroup model’s approach rather than the more efficient approach demonstrated by an outgroup model. Thus, although some work on imitation in China has been reported: (i) no study to date has examined children’s imitation of novel acts in an over-imitation test paradigm, and (ii) no study in China has tested the effects of pedagogical cues and prior self-experience.

Another potential contribution is that we used a broader range of measures of imitation than have typically been used in studies of preschool imitation. We measured: (i) the duplication of the overall outcome or end-state of the adult demonstration, (ii) high-fidelity imitation of novel target acts performed en route to achieving this end-state, and (iii) the duplication of the correct serial order of these novel target acts. These multiple measures help to illuminate the scope and functions that imitation may serve in human childhood. For example, if childhood imitation is a mechanism by which culturally specific rituals and customs are acquired (e.g., Rossano, 2012; Legare and Nielsen, 2015; Legare, 2017), children would need to be attentive to and capable of imitating the serial order of behaviors (Loucks et al., 2017), because rituals often demand duplicating the order in which arbitrary acts are performed (e.g., chanting before drinking the wine). In sum, the results of the current experiment promise to expand our knowledge about the factors modulating imitation of novel acts and their serial order in preschoolers beyond those in Western culture, and thereby enrich our understanding of the functions, value, and scope of imitation in *Homo sapiens*.

## Materials and Methods

### Participants

We tested a large sample of children in China. A total of 210 children ages 4 years (110 males; *M*_age_ = 51.74 months, *SD* = 4.78 months) were recruited for this study. Children were recruited from a preschool in Xuzhou city, a mid-sized town in Jiangsu Province in the eastern part of mainland China. All participants were of Han ethnicity. The study was approved by the ethics committee of Jiangsu Normal University and the procedures were carried out in accord with this approval. Written parental permission for school testing of each child was obtained, and children received a small reward for their participation (e.g., stickers).

### Test Environment, Design, and Materials

Children were tested individually in a separate room at their school that contained a small table for the child and experimenter (a female, native Chinese) to sit at for the test session. Each child was randomly assigned to one of five independent groups, with *n* = 42 children in each group. Within each group, children were randomly assigned in terms of (i) child’s gender and (ii) order of the test objects (ABCD, BCDA, CDAB, and DCBA).

**FIGURE 1:**
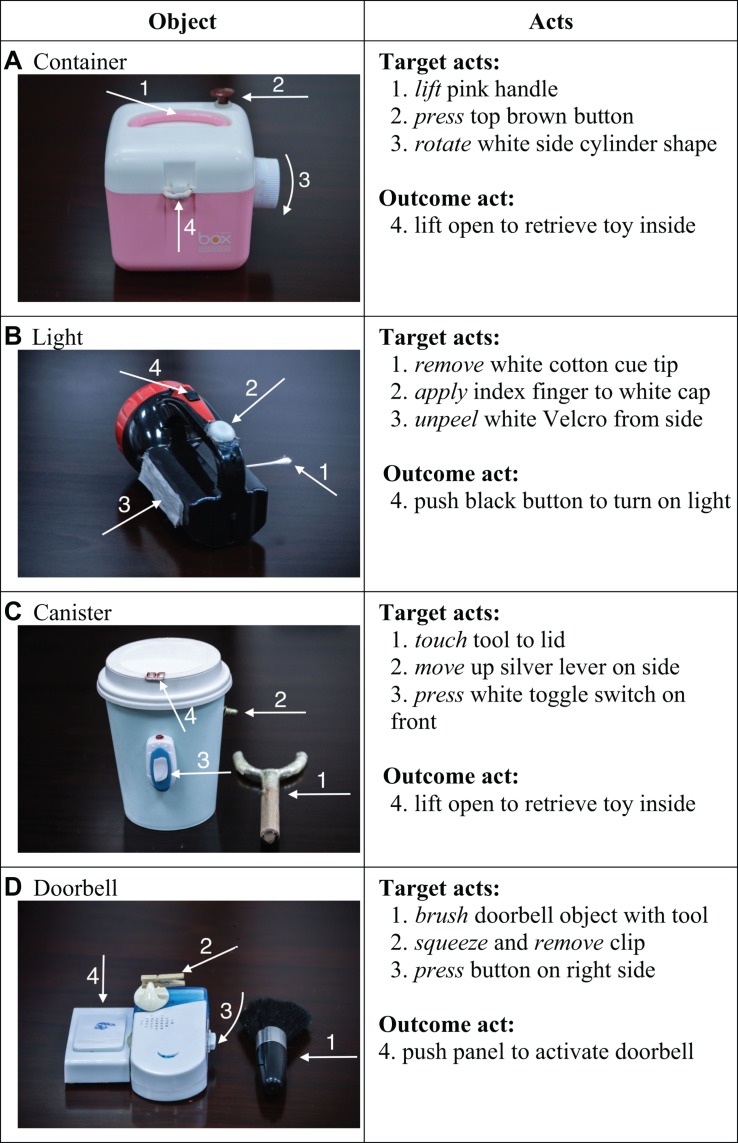
Photographs of the four test objects **(A–D)**, as well as verbal descriptions of the three novel target acts and the final outcome act for each object. See Figure 2 for human behaviors.

Four novel objects were manufactured based on previously published work (Gonsiorowski et al., 2016). We combined several elements together to manufacture new objects, with the dual goals of (i) making the materials look somewhat unfamiliar to the children to heighten interest and (ii) assembling materials that allowed us to perform novel acts that the children would be unlikely to have seen or performed in the past (with the goal of making them relatively low-baseline acts). For example, we employed a small brush to stroke a doorbell for no apparent reason. Figure 1 displays the collection of objects and provides a description of the target acts. For each object, the experimenter performed three novel and unnecessary acts (hereafter “target acts” because these are used to measure the ability to imitate novelty) before demonstrating a final act that caused the desired outcome. A video camera was used to record the study for subsequent scoring.

### Procedure

**FIGURE 2:**
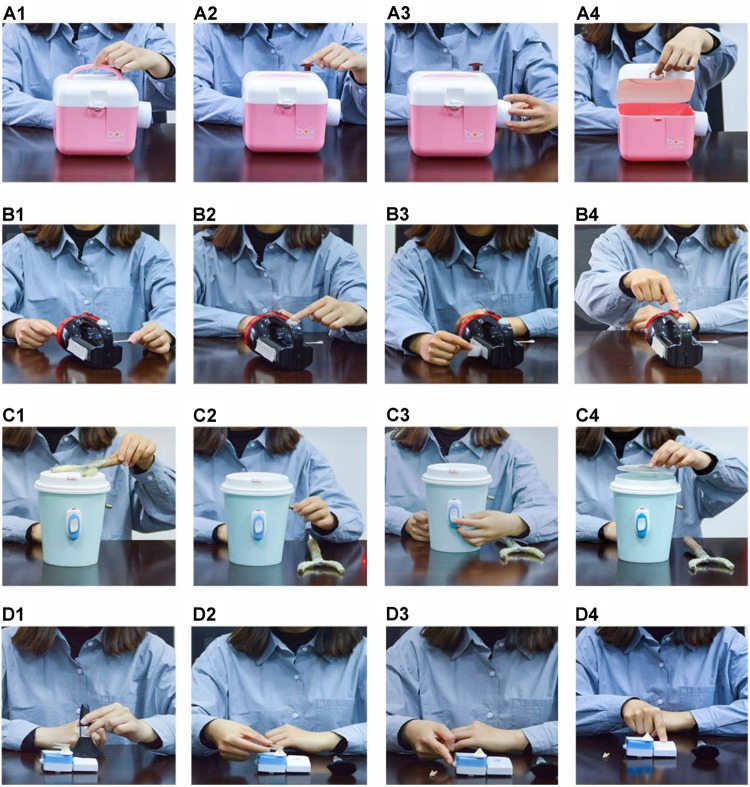
Photographs of the four test objects (shown as the rows **A1–D4**). Within each row, photos show the three arbitrary target acts (Acts 1–3, in each row) and the outcome act that leads to a salient outcome (Act 4, in each row). See also Figure 1 and main text for more details.

In the four treatment groups (Groups 1–4), children saw the adult demonstrations of the three novel target acts and the final outcome act. To an adult observer, the three target acts were not causally necessary to achieve the outcome. For example, the adult demonstrated the novel act of brushing a doorbell with a women’s makeup-brush (designed to apply powder to the cheeks) prior to demonstrating the act of ringing the doorbell. We cannot be sure that the children construed the brushing act as non-causal or irrelevant to doorbell ringing, but it is justifiable to call it “novel” or arbitrary, because children have not seen someone brush a doorbell it in the past and have not been trained to perform this specific act. Each of the three novel target acts could be executed independently of one another and in any order, and were not needed to achieve the final outcome act (see Figure 2 for demonstrated behaviors). For each test object, the adult demonstrated the three target acts before performing the final outcome act.

The fifth independent group of children (Group 5) served as a baseline control in which the children received no adult demonstration before being presented with the object. This group assessed the probability that the children in the response period would spontaneous produce the target or outcome acts, in the absence of seeing them demonstrated.

#### Demonstration Phase

Table 1 provides an overview of the manipulations used in each of the five the independent groups used in the experiment. Procedural details are described below.

**TABLE 1:** Description of each of the five groups.

**Group**	**Demonstration**	**Prior**	**Pedagogical**	***n***
		**experience**	**cues**	
(1) Demo(−prior +ped)	**+**	**−**	**+**	42
(2) Demo(−prior −ped)	**+**	**−**	**−**	42
(3) Demo(+prior +ped)	**+**	**+**	**+**	42
(4) Demo(+prior −ped)	**+**	**+**	**−**	42
(5) Control (baseline)	**−**	**−**	**−**	42

**N = 210 total. Demo, adult demonstration; ped, pedagogical cues*.*

##### Group 1: demo(−prior +ped)

Children in this group saw the adult demonstration (indicated by the word “Demo”). They observed this demonstration along with pedagogical cues (indicated by “+*ped*”) and without having any prior self-experience handling the test object (indicated by “−*prior*”). The experimenter smiled and made eye contact with the child and the tone of voice of the experimenter was warm and friendly as if “showing” or teaching the action to the child (pedagogical cues). The experimenter drew the child’s attention by saying, “Today we are going to play a game. It’s my turn first. Then it will be your turn.” The experimenter performed the three novel target acts and then the outcome act of opening the lid to obtain the toy inside. After the demonstration, the experimenter removed the object from the children’s view and reset it to the starting state and the children were handed the object for the 30-s response period (see below). Next, she re-established eye contact with the child and began a new demonstration with the next object until she had completed the demonstrations with the four test objects in one of the randomly assigned orders (e.g., ABCD).

##### Group-2: demo(−prior −ped)

The procedure in this group was the same as Group-1, except that the pedagogical cues were removed. Specifically, the experimenter did not smile or make eye contact with the child. When the experimenter said a sentence, the experimenter’s tone of voice was neutral. During the demonstration, the experimenter’s eyes remained fixed on the object rather than making eye contact with the child first.

##### Group-3: demo(+prior +ped)

The procedure was the same as Group-1 except that an initial self-experience period was added. During the self-experience phase, children were allowed to play with each object; specifically, the experimenter placed the object in front of the child and said, “Go ahead, you play with it first.” The duration of the self-experience period was a fixed 30 s, electronically timed. After this interval was complete, the experimenter asked for the object, removed it from view, and then re-presented it to the physical starting state was the same as Groups 1 and 2, and said, “Now it is my turn to play with it, Look.” The remainder of the procedure was identical to Group-1.

##### Group-4: demo(+prior −ped)

The procedure was the same as Group-1 except that the pedagogical cues were removed, and the prior self-experience handling the object was added.

##### Group-5: control (baseline)

The demonstration phase was skipped for children in this group. They were administered the response period only, as described in the next section.

#### Response Period

The response period was the identical for all five groups. For all children, the identical protocol was followed: The adult simply handed each the object to the child to play with for an electronically timed 30-s period.

### Dependent Measures and Behavioral Coding

#### Target Act Score

There were three novel target acts for each object (Figures 1, 2). Children obtained one point for each target act they performed on each object during the 30-s response periods. Thus, for each child, the target act scores ranged from 0 to 12 (4 objects × 3 target acts).

#### Serial Order Score

The three novel target acts for each object were demonstrated in a serial order (Act 1 → Act 2 → Act 3). For each object, the child could copy the three target acts in the full correct order (1-2-3), or s/he could copy only two of the three target acts in the correct order (1-2, 2-3, or 1-3). The serial order score was a dichotomous 0 or 1 for each object. If the child copied any correctly ordered pair of acts (1-2, 2-3, or 1-3) or the entire sequence 1-2-3 for an object, s/he was scored as a 1. If not, s/he was scored as a 0. Thus, the total serial order score ranged from 0 to 4 (maximum score of “1” × 4 objects = 4).

#### Outcome Act Score

The child received a 1 if he or she reproduced the final outcome act for each object. Thus, the scores ranged from 0 to 4 (maximum score of “1” × 4 objects = 4).

#### Coding Agreement

The primary scorer was a research assistant who remained uninformed of the participant’s group assignment and the study hypotheses. A second scorer, also unaware of group assignment, coded a randomly selected 25% of the participants. Intercoder agreement was assessed by Cohen’s kappa and was high for all the dependent measures (target act, *k* = 0.96; serial order, *k* = 0.92; outcome act, *k* = 0.99).

## Results

Preliminary analyses showed no significant effects of gender or object presentation order on any of the dependent measures, and thus the data were collapsed across these factors for subsequent analyses.

There was strong evidence for imitation. A one-way analysis of variance (ANOVA) was conducted using each of the three dependent measures, and the results showed that each significantly varied as a function of the experimental groups (Table 2). There was a significant effect for children’s *target act score*, *F*(4,205) = 37.76, *p* < 0.00001, $\eta_{\text{P}}^{2}$ = 0.65. Follow-up comparisons (LSD) showed that children in the Demo(−prior +ped) group (*M* = 10.26), i.e., children who had no prior experience, performed significant more target acts than did children in each of the two groups that had prior experience: Demo(+ prior +ped), *M* = 8.95, *p* = 0.009, and Demo(+prior −ped), *M* = 8.90, *p* = 0.007. Moreover, there was no significant difference between the two groups with no prior experience: Demo(−prior +ped), *M* = 10.26, and Demo(−prior −ped), *M* = 9.36, *p* = 0.069.

**TABLE 2:** Mean (*SD*) of dependent measures as a function of test group.

	**Target acts**		**Serial order**		**Outcome act**	
			
**Groups**	***M***	***(SD)***	***M***	***(SD)***	***M***	***(SD)***
Demo(**−**prior +ped)	10.26	(1.59)	3.24	(1.03)	3.33	(1.18)
Demo(**−**prior **−**ped)	9.36	(2.32)	2.98	(1.07)	3.50	(0.74)
Demo(+prior +ped)	8.95	(2.91)	2.43	(1.25)	3.60	(1.01)
Demo(+prior **−**ped)	8.90	(2.06)	2.48	(1.23)	3.67	(0.79)
Baseline control	4.71	(2.28)	0.43	(0.83)	2.62	(1.32)

**Demo, adult demonstration;****−****prior, no prior self-experience; +prior, with prior self-experience;****−****ped, no pedagogical cues; +ped, with pedagogical cues.**

There was also a significant effect as a function of group for children’s *serial order score*, *F*(4,205) = 42.82, *p* < 0.00001, $\eta_{\text{P}}^{2}$ = 0.68. Follow-up comparisons showed that children in the Demo(−prior +ped) group (*M* = 3.24), i.e., children who had no prior experience, had significant higher serial order scores than did children in each of the groups that had prior experience: Demo(+prior +ped), *M* = 2.43, *p* = 0.001, and Demo(+prior −ped), *M* = 2.48, *p* = 0.002. Moreover, there was no significant difference between the two groups with no prior experience: Demo(−prior +ped), *M* = 3.24, and Demo(−prior −ped), *M* = 2.98, *p* = 0.274.

As expected, there was also a significant effect as a function of group for the *outcome act score*, *F*(4,205) = 7.04, *p* < 0.00003, $\eta_{\text{P}}^{2}$ = 0.35 with each treatment group (Groups 1–4) having significantly higher scores than the Control (baseline group), all *p*s ≤ 0.001.

**FIGURE 3:**
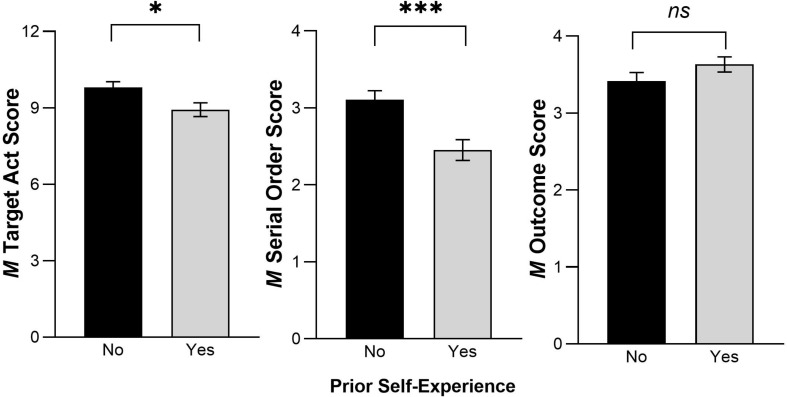
Children’s responses as a function of having or not having prior self-experience for each dependent measure. Error bars show ±1*SE. ^∗^p* < 0.05, ^∗∗∗^*p* < 0.001.

To provide a further statistical probe, we also conducted planned comparisons among the four treatment groups (Groups 1–4) to assess the effects of prior self-experience and pedagogical cues. A 2(Prior self-experience: yes vs. no) × 2(Pedagogical cues: yes vs. no) ANOVA was conducted on each dependent measure. For the target act score, as predicted (Williamson et al., 2008; Wood et al., 2013), there was a significant main effect of prior self-experience, *F*(1,164) = 6.34, *p* = 0.013, $\eta_{\text{P}}^{2}$ = 0.037: When children did not have prior self-experience with the objects (*M* = 9.81) they produced significantly more of the novel target acts than when they had prior experience handling the objects (*M* = 8.93), (Figure 3). There was no significant main effect of pedagogical cues, *F*(1,164) = 1.85, *p* = 0.175, and there was no prior experience × pedagogical cues interaction, *F*(1,164) = 1.50, *p* = 0.222.

For the serial order score, there was also a significant main effect of prior self-experience, *F*(1,164) = 13.59, *p* < 0.0004, $\eta_{\text{P}}^{2}$ = 0.077, showing that children who had no prior self-experience (*M* = 3.11) were significantly more likely to imitate the serial ordering of the behaviors they observed than children with prior self-experience (*M* = 2.45) (Figure 3). There was no significant main effect of pedagogical cues, *F*(1,164) = 0.36, *p* = 0.547, and no prior experience × pedagogical cues interaction, *F*(1,164) = 0.76, *p* = 0.385.

For the outcome act score, there was no significant main effect of prior self-experience, *F*(1,164) = 2.15. *p* = 0.145 (Figure 3), and also no significant main effect of pedagogical cues, *F*(1,164) = 0.66, *p* = 0.417, and no prior experience × pedagogical cues interaction, *F*(1,164) = 0.11, *p* = 0.745. These results are informative when considered in the context of the larger pattern of results. More specifically, they show that even though children re-enacted significantly more of the causal outcome acts when they saw them modeled in the four Demonstration groups (Groups 1–4) than they did in the Control (baseline) group (see Table 2 and statistical results reported above), the children produced these causal outcome acts regardless of whether or not they had prior experience or pedagogical cues (possibly because these acts led to a physical outcome or reward of finding the toy). However, there *was* significant modulation of imitation as a function of prior self-experience for imitation of the non-causal, novel target acts in the same children in the same experiment. These novel acts did not have to be performed by the child to reach the instrumental end of finding the toy.

## Discussion

The current study extends efforts to understand the nature and scope of imitation in children who are not from Western, educated, industrialized, rich democratic societies. This effort is partially motivated by the desire to determine which aspects of imitation may be more culturally invariant and which are more variable and dependent on cultural context. A range of topics within social learning, using different paradigms, have been tested using non-Western samples (e.g., Itakura et al., 2008; Nielsen and Tomaselli, 2010; Nielsen et al., 2014; Berl and Hewlett, 2015; DiYanni et al., 2015; Wang et al., 2015; Clegg and Legare, 2016; Corriveau et al., 2017; Taniguchi and Sanefuji, 2017; Hoehl et al., 2019; Li et al., 2019). However, little research has investigated the imitation of preschool children born and raised within China, a country that more than 1 billion people (for exceptions see Wang et al., 2015; Li et al., 2019). Children raised in China tend to be socialized in ways that are distinct from Western cultures, and also from many of the non-Western cultures tested in the studies referred to above. The present work is the first to systematically test how two prominent factors reported to modulate imitation in Western children—pedagogical cues and prior self-experience—influence children’s imitation in China.

One aim of the current study was to investigate whether preschool Chinese children’s imitation of novel acts varied as a function prior self-experience. We found that such experience significantly influenced high-fidelity imitation of specific novel target acts demonstrated by the adult. These finding are in line with studies of self-experience involving Western children (Williamson et al., 2008; Williamson and Meltzoff, 2011; Wood et al., 2013; Schleihauf et al., 2018).

We offer the speculation, based on these results and extant theory, that the influence of prior self-experience may be (relatively) culturally invariant, although more research is needed across a wider range of cultures. We theorize that invariance across cultures makes adaptive sense for the prior experience factor, because it draws on what the child extracts from manipulating the object on their own—the consequences of self-actions and self-agency, which is part of play behavior across the world. Through exploring the object themselves, children often develop their own successful routines, procedures, and conceptions of how to use the object. In certain contexts this can diminish the high-fidelity imitation of novel, meaningless, and irrelevant acts demonstrated by others. This pattern of findings can be linked to theories of education. Children are agents who acquire knowledge by self-directed exploration the physical and social world. The value of young children’s play and joyful feelings self-agency is often emphasized in early education (Bruce, 2018; Delafield-Butt, 2018; Trevarthen, 2018). Some psychologists and educators have suggested that in the preschool classroom, it is conducive to allow children to become more active agents by purposely diminishing the adult’s own activity and authority (Montessori, 1966; Trevarthen et al., 2018). The power of play for engendering creative interactions with objects was originally emphasized by Vygotsky (1978) as well as by Piaget (1952, 1962) who contrasted the child’s drive for exploration and assimilation (play) with that of accommodation to others (imitation). Ultimately, the engine for human development and learning is fueled by both play and imitation. Children need to combine first-person, hands-on experiences (play) and those experiences gained from third-party observation of the acts of other people (imitation). Modern, effective preschool education can strive to foster children’s adaptive ability to integrate these activities according to the social, emotional, and cognitive goals and contexts at hand.

Another aim of the current study was to examine the degree to which Chinese children’s imitation was influenced by pedagogical cues. Results showed that native Chinese children reproduced the novel target acts at approximately the same levels regardless of whether these acts were demonstrated with or without the support of pedagogical cues. This finding does not fit easily with the predictions from the theory of natural pedagogy (Gergely et al., 2007; Csibra and Gergely, 2009). According to this idea, at least in its strongest form, pedagogical cues indicate to children that the adult is teaching cultural knowledge about how to use the object which may engender, or at least enhance high-fidelity imitation of novel acts. However, native Chinese children did not respond in a significantly different way to demonstrations with pedagogical cues versus seeing those same acts demonstrated without the pedagogical cues. This restricts the scope of theories about pedagogical cues and suggests they may be more applicable to children reared in Western rather than traditional Chinese culture (or perhaps play a greater role in children at a different age than those tested here). Going one step further, the current results align well with emerging findings reporting that young Western children can copy modeled actions when no pedagogical cues are present (Schmidt et al., 2011; Shimpi et al., 2013; Hoehl et al., 2014). It is thus possible that pedagogical cues are not as *necessary* as a strong view would predict, but may elevate the expression of imitation in Western children under particular circumstances. Further research on Western children varying the age/developmental level and the specific tasks used (Yu and Kushnir, 2014, 2020) may bring further clarity to these issues, but they are beyond the scope of the current research.

The proclivity of human children to imitate novel, non-causal, “meaningless” acts as well as their serial order is noteworthy. Non-human primates are capable of duplicating outcomes or end-states (such as opening a container to retrieve an edible piece of food, sometimes called “goal emulation”), but they less readily engage in high-fidelity imitation of the arbitrary novel acts and mannerisms of a model when they have no physical-causal significance or rewarding outcomes (Hoehl et al., 2019; Tomasello, 2019). It has also been reported in children with autism spectrum disorder (ASD) show more deficits in imitating of the specific behaviors and arbitrary mannerisms of adult models than in achieving the demonstrated outcomes or end-states through other means (for a meta-analysis of imitation in children with ASD see Edwards, 2014; see also Toth et al., 2006; Nadel, 2014).

The tendency of typically developing human children to imitate the details of arbitrary novel acts with high fidelity, as shown in the current study, fits hand-in-glove with the uniquely human characteristic of diverse and cumulative culture (Legare, 2017; Meltzoff and Marshall, 2018; Tomasello, 2019). Such high-fidelity imitation enables children to acquire complex behaviors that they are unlikely to hit upon by themselves (e.g., Yu and Kushnir, 2014; Subiaul et al., 2015). Moreover, high-fidelity imitation of the serial order of novel acts (also documented here) is especially well-suited for the intergenerational transfer of culturally specific customs and rituals. To enact rituals, one needs to do specific behaviors in the right sequence for it to “count.” In Western cultures, the incantation in church must precede the sip of wine—not the reverse. Likewise, in a prominent Chinese Buddhist worship ritual, one chants scriptures, makes a kowtow, and then inserts the incense into the center of the alter. One does not insert the incense first and then make a kowtow.

We established that children not only imitated novel, arbitrary acts, but also that they tended to repeat these acts in the same serial order in which they witnessed them, and they exhibited significantly higher levels of such novel imitation when they did not have prior self-experience with the objects that would have led them to manipulate the objects in other ways. Interestingly, “sacred objects” are often kept quarantined and saved for ritualistic occasions, not usually handled in ways that conflict with the ritual. In adult rituals (and perhaps to a lesser extent the novel acts in this study), the “meaning” of the sequence of witnessed acts does not derive from the fact that they cause an immediate, physically contingent outcome or reward, but from the fact that the whole ritual—including the serial order of the acts (the chanting then the kowtowing then the placing of incense)—takes on social meaning.

Together, these findings suggest that future theoretical effort should be devoted to how children learn both the sociocultural conventional and non-conventional uses of objects by interweaving their observation of others together with their own personal history with the objects and actions. One emerging perspective, dubbed the “socio-materiality” viewpoint (Iannaccone, 2015) has begun this examine this complex interaction between people (self/other), objects, and cultural meanings to assess how they interact in social-cognitive development. This fundamental issue also animated the work of Piaget (1962) and Vygotsky (1978) and is increasingly informing modern perspectives on early education (Master et al., 2017; Trevarthen et al., 2018).

### Limitations, Future Directions, and Conclusion

The current study is not without limitations. First, we tested children using the standard procedure of having the adult model remain present during the child’s response period. This is common with studies of 4-year-old children because it is not so easy to leave them alone and unattended in a room. It is possible that children in China regard the model as an authority figure or teacher who they should conform to (her presence might provide a motivation to perform the act, although she remained present in *all* of the groups tested, and thus the differences between groups cannot be attributed to this). It would thus be interesting to design future experiments of prior self-experience and pedagogical cues, while experimentally manipulating whether the model did or did not remain present watching the child’s actions (for studies on the role of the presence of the experimenter, see e.g., Hanna and Meltzoff, 1993; Klein and Meltzoff, 1999; Repacholi and Meltzoff, 2007; Nielsen and Blank, 2011; Hoehl et al., 2014).

Second, it would be useful to use the identical experimental procedures in both China and the United States. At present, we can only draw loose inferences about (Western) pedagogical cues not having as strong an effect in children born and raised in China as they do in Western cultures. This is because the various studies evaluating pedagogical cues in Western cultures have used different ages, procedures, and/or objects from each other, and so strict cross-cultural comparisons are difficult. Our primary aim was to investigate how these factors influence imitation in China, a country encompassing more than 1 billion people and of interest to theory because of the value placed on group cohesion, harmony, and conformity, and a different pattern of child-rearing practices than Western cultures, which could influence young children’s social and “other-directed” behavior (e.g., Barragan et al., 2020). A controlled comparison to Western samples using this same paradigm and age was beyond the scope of this paper.

Third, our inferences are limited to the broad but delimited set of objects and tasks that we tested. We used a range of objects (four) and a range of acts (three novel acts plus one goal-directed causal outcome act), but there are many other different types of demonstrations that are also of interest (e.g., tool affordances; variations in the causal opacity of the acts; reliability, trustworthiness, and efficacy of the model; manipulations that vary the motivation to affiliate with the adult or conform to cultural norms; demonstrations by ingroup vs. outgroup models, etc.). We are not making the claim that the factors explored here are the *only* factors that modulate preschool imitation. Further research could be conducted that pits prior self-experience and pedagogical cues against one or more of these other foregoing factors, both within and across cultures, to further examine cultural variations in factors that govern childhood imitation of novel acts.

### Broader Theoretical Implications About Imitation, Culture, Mind

Continued research is warranted on factors that modulate preschool children’s high-fidelity imitation of novel acts. The diverse and cumulative aspects of human culture—widely celebrated by evolutionary biologists and psychologists (e.g., Henrich and McElreath, 2003; Legare, 2017; Tomasello, 2019)—crucially depends on learning novel acts through observation and imitation from others in the cultural milieu (Meltzoff and Marshall, 2018). Importantly, young human children can and do imitate novel acts in situations in which people are not intentionally teaching them. Imitation is a powerful mechanism for the intergenerational transfer of behaviors, skills, customs, and norms, based purely on observation of the acts of others, even in the absence of those people’s conscious efforts to teach. Children around the world and in all cultures learn from observing and imitating others; however, what they imitate, who they imitate, and when they imitate is malleable. By further understanding what motivates and modulates imitation, we will enhance our understanding of mind, culture, and social learning.

## Data Availability Statement

The datasets generated for this study are available on request to the corresponding author.

## Ethics Statement

The studies involving human participants were reviewed and approved by ethics committee of Jiangsu Normal University. Written informed consent to participate in this study was provided by the participants’ legal guardian/next of kin.

## Author Contributions

ZW and AM conceptualized the experiments. ZW designed the methodology, performed the experiments, and analyzed the data. ZW and AM wrote the manuscript.

## Conflict of Interest

The authors declare that the research was conducted in the absence of any commercial or financial relationships that could be construed as a potential conflict of interest.
